# Can Henna Prevent Ulceration in Diabetic Feet at High Risk?

**DOI:** 10.1155/2009/107496

**Published:** 2009-11-25

**Authors:** Mesut Mutluoğlu, Günalp Uzun

**Affiliations:** Department of Underwater and Hyperbaric Medicine, Gülhane Military Medical Academy, Haydarpaşa Teaching Hospital, Üsküdar, 34668 İstanbul, Turkey

Diabetic foot infections are one of the most dreadful and costly complications of diabetes. Efforts have recently focused on preventive measures. The balance of moisture in the neuropathic foot is critical and challenging. Whilst the interdigital areas should be kept dry to prevent maceration and subsequent softening and thinning of the skin other areas of the foot should be managed for optimal moisture to avoid dry skin and resulting cracks. 

Oftentimes, the primary cause of infection is a breakdown of skin integrity. Several factors may cause a break in healthy skin, however some parts of the foot, notably the interdigital area, are more prone to compromise. The interdigital area, which usually provides entrance points for infectious agents, is a rather closed environment and may therefore be subject to more moisture than any other part of the foot. Excess moisture may in turn cause maceration which increases the likelyhood of skin breakdown and microbial colonisation and invasion. Indeed, it has been shown that the skin in interdigital areas of the diabetic patients has higher PH levels which promotes susceptibility to candidal infections [1]. 

Many products have been developed for the protection of skin integrity. Topical henna is an extract of the lawsonia plant. Some experimental and clinical studies have reported antibacterial and antifungal effectiveness and wound healing activity of this product [2–4]. Henna is an easily accessible, inexpensive product which, to our best knowledge, has not been studied so far in the diabetic foot at risk. The henna extract may be prepared by mixing 1 gr of powdered leaves to 10 mL of distilled water which will subsequently be applied to the interdigital space. It is then kept wrapped with a dressing for 4–6 hours before it is washed. This procedure will provide a long-term barrier against moisture in the problematic interdigital area (1 to 3 months) and thus will not require frequent application. Henna may also improve wound healing in fissures and cracks in diabetic feet. 

As prevention is of paramount importance, studies should focus more on intensive preventive strategies that are promising in maintaining healthy skin. In this context, henna could be a valuable, inexpensive, and readily accessible product that does not require frequent application to maintain skin integrity in the interdigital area.
